# Enhanced nitrate removal from aqueous solutions using amine-functionalized biowaste-derived adsorbent

**DOI:** 10.1038/s41598-025-17259-9

**Published:** 2025-10-21

**Authors:** Tiantian Li, Lang Liu, Meng Li, Ying Li

**Affiliations:** 1 College of Energy Engineering, Xi’an University of Science and Technology, Xi’an, 710054 China; 2 Key Laboratory of Western Mine Exploitation and Hazard Prevention, Ministry of Education, Xi’an, 710054 China

**Keywords:** Nitrate removal, Biobased adsorbent, Amine functionalization, Electrostatic interactions, Environmental chemistry, Pollution remediation

## Abstract

Nitrate ions constitute one of the major nitrogen resources for plant growth. However, the large amount of nitrate ions released into nearby waterbodies could cause eutrophication problems. This study developed a bio-based adsorbent from biowaste materials and functionalized it with amine groups through the Mannich reaction. The successful modification was confirmed using FTIR, SEM, and elemental analysis. Batch adsorption experiments were conducted under varying conditions, including contact time, pH, and initial nitrate concentration. Results showed that the amine-functionalized bio-adsorbent exhibited an enhanced capacity for nitrate removal, with a maximum adsorption capacity of 65.79 mg g^−1^. Electrostatic interactions between amine-functionalized bio-adsorbent and nitrate ions were identified as the dominant sorption mechanism. These findings highlight the potential of this bio-based adsorbent as a sustainable and effective solution for nitrate removal from contaminated water sources.

**Supplementary Information:**

The online version contains supplementary material available at 10.1038/s41598-025-17259-9.

## Introduction

Nitrate (NO_3_^−^) is a primary nitrogen source for plant growth^1^. However, excessive release of nitrate ions into water bodies can result in significant environmental and human health issues, such as eutrophication, methemoglobinemia and the production of carcinogenic nitrosamines and nitrosamides^1–3^. To prevent these issues, many technologies have been developed to remove nitrate ions from water, such as biological treatment, chemical precipitation, reverse osmosis, and electrodialysis^4–6^. Despite their effectiveness, these methods have notable limitations. For example, reverse osmosis and electrodialysis involve high operational costs, whereas biological denitrification requires specific microorganisms, controlled temperature conditions, and additional posttreatment processes. In contrast, adsorption has emerged as a promising alternative due to its cost-effectiveness, simple design, and ease of operation^7^. But its performance is highly dependent on the physicochemical properties of the adsorbent material utilized^2^.

Various materials, such as polymeric resins and activated carbon, have been studied and developed as adsorbents for removing nitrate ions from water^8–10^. However, when considering the downstream use of recovered nitrogen as fertilizer in agricultural systems, biomass-derived waste materials have emerged as ideal candidates because their low cost, biodegradability, and minimal contamination risks^11–13^. Among these, the lignin has received growing attention in recent years, owing to its abundance, renewability, and unique phenolic macromolecular structure^14–16^. Lignin is one of the most plentiful sources of aromatic compounds in nature, and its highly branched phenolic backbone makes it a promising platform for the synthesis of value-added chemicals, bio-based materials, and fuels^17–19^. Beyond its structural utility, lignin can also contribute to agronomic functions^20–23^. For example, it is considered a precursor to humic acids that can be transformed by soil microorganisms, thereby enhancing soil organic matter and fertility^24^. Moreover, lignin has been shown to modulate soil nitrogen cycling by inhibiting urease activity, which slows urea hydrolysis and reduces nitrogen losses^25^.

Despite these advantages, lignin exhibits limited nitrate adsorption capacity because it lacks strongly positive adsorption sites^1,15^. To address this limitation, chemical and physical surface modifications have been explored to enhance nitrate adsorption efficiency. Several surface modification methods, including protonation, metal impregnation, and amine grafting, have been investigated^1,2^. Among these techniques, amine grafting has recently gained much attention due to its effectiveness in significantly enhancing nitrate removal efficiency. By introducing positively charged amine groups onto the lignin surface, this method significantly promotes adsorption of negatively charged nitrate ions through electrostatic interactions. Furthermore, amine grafting increases the nitrogen content of lignin, which reduces its carbon-to-nitrogen (C/N) ratio, accelerating biodegradation and enhancing its efficacy as a slow-release nitrogen fertilizer in agriculture soils^21,26^.

Urea is a widely used, cost-effective, and low-toxicity nitrogen source essential for promoting plant growth. It also serves as a versatile precursor for synthesizing nitrate rich materials, such as graphitic carbon nitride(g-C_3_N_4_)^27^. Given its affordability, wide availability, and environmentally benign nature, urea is considered a sustainable amine source for increasing the nitrogen content of materials through functionalization. In this study, we propose a facile and environmentally friendly method for preparing amine-functionalized lignin through the Mannich reaction, eliminating the need for high-pressure conditions or external catalysts. Specifically, the Mannich reaction directly incorporates amine groups into the lignin structure through a straightforward C–N bond-forming step. Initially, reactive hydrogen atoms of the amine groups in urea react with formaldehyde (HCHO) to form imine intermediates, which subsequently bond to phenolic guaiacyl units within lignin, resulting in amino-alkyl functionalization of the lignin side chains^28^. After amine functionalization, the morphological and structural changes of lignin were characterized through Fourier transform infrared (FTIR) spectroscopy, scanning electron microscopy (SEM) and elemental analysis. The nitrate adsorption performance of resulting amine-functionalized lignin was evaluated under various experimental conditions, including initial concentration, contact time, and pH. Finally, the adsorption mechanism was discussed, and adsorption behaviors were analyzed by fitting experimental data to different isotherm and kinetic models. This study not only suggests a novel approach for the integrated utilization of lignin resources but also provides a potentially efficient and eco-friendly adsorbent for the recovery of nitrate and the treatment of contaminated water.

## Materials and methods

### Chemicals

Lignin, urea and formaldehyde were purchased from Fisher Scientific (Pittsburgh, Pennsylvania), and calcium nitrate was obtained from Sigma Aldrich (St. Louis, Missouri). NitraVer^®^ 5 Nitrate Reagent Powder Pillows were purchased from Hach company (Loveland, Colorado, USA). Chemical reagents of analytical grade, including sodium hydroxide (NaOH) and hydrochloride acid (HCL), were purchased from Fisher Scientific. All chemicals used in this study were ACS grade from Fisher Scientific (Hampton, NH) or Sigma Aldrich (St. Louis, Missouri).

### Preparation of amine-functionalized lignin

**Fig. 1:**
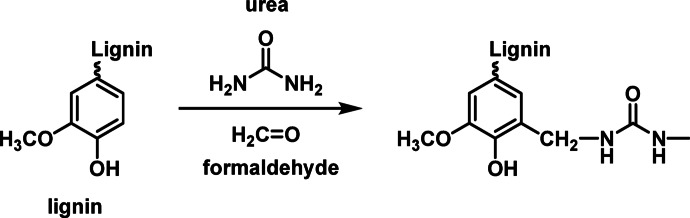
Synthetic reactions for amine-functionalized lignin.

Amine-grafted lignin was synthesized through the Mannich reaction, as illustrated in Fig. 1. Briefly, 20 g of lignin was placed in a 250-ml single-neck glass flask before 100 ml of distilled water was added to the single-neck flask. The solution in the flask was subsequently heated on a hot plate at 50 °C. The solution pH was adjusted to 11 by using 1 M NaOH. Then, 3 g of urea and 1.95 ml of formaldehyde were added to the flask, and the mixture was incubated at 90 °C for 4 h. After the reaction, the mixture was cooled to an ambient temperature, and 1 M (10%) HCl was carefully added until the precipitates formed. The precipitates were filtered through filter paper and washed with distilled water until the pH reached neutral.

Finally, the amine-functionalized lignin was dried at 65 °C under vacuum for 24 h. The final products were dark brown and were ground to powders for use.

### Characterization of amine-functionalized lignin

Fourier transform infrared spectroscopy (FTIR) spectra were recorded on a Magna System 560 from Nicolet Instrument (WI, USA). Sample powders were obtained after grinding with potassium bromide (KBr) (1% w/w to KBr) before testing. Each sample was measured via 64 scans at a resolution of 4 cm^− 1^ and in diffuse reflection mode. The abundances of C, N, and H were determined by a CHN Elemental Analyzer (Carlo-Erba NA-1500) through high-temperature catalyzed combustion followed by infrared detection of the resulting CO_2_, H_2_ and NO_2_ gases, respectively. The surface states of lignin were characterized by scanning electron microscopy (SEM), which was performed with an FEI Nova NanoSEM 430 (USA, operating voltage of 30 kV).

### Batch adsorption test

In each run, 25 mg of amine-functionalized lignin or unmodified lignin was added to 40 ml of calcium nitrate solution at various concentrations ranging from 50 mg L^− 1^ to 500 mg L^− 1^. The mixtures were subsequently placed in a mechanical shaker and shaken overnight. After being shaken overnight, the solution was filtered through 0.22 μm syringe nylon-membrane filters. The equilibrium nitrate concentration (C_e_, mg L^− 1^) in the filtrates was analyzed according to the nitrate chromotropic acid TNT method with NitraVer^®^ 5 nitrate reagent powders by a DR-900 colorimeter (HACH Company, Loveland, Colorado, USA) at 420 nm.

#### Effects of pH and contact time

The effect of pH on the nitrate adsorption capacity was investigated with an initial nitrate concentration of 100 mg L^− 1^ at pH values ranging from 3 to 10. The pH of each solution was adjusted by carefully adding diluted NaOH or HCl, and the pH was measured with a pH meter until it stabilized. Then, 25 mg of adsorbent was added to 40 ml of nitrate solution with different pH values. After shaking overnight, the solutions were filtered, and the equilibrium nitrate concentrations in the filtrates were analyzed as described previously. For experiments on the effects of contact time, the rate of adsorption of nitrate was determined in a flask containing nitrate solution with 200 mg L^− 1^ at the room temperature. The sample was withdrawn and filtered at different time intervals to determine the variability in nitrate concentration with time.

#### Adsorption kinetics and isotherm study

To gain deeper insight into the adsorption mechanism of nitrate ions onto amine-functionalized lignin, several isotherm models, including the Langmuir, Freundlich, Temkin, and Redlich–Peterson models, were applied to fit the experimental data. For the adsorption kinetics experiments, the rate of adsorption of nitrate was determined in a flask, and the concentrations of nitrate at different time intervals were measured as described in the previous section. To evaluate the adsorption kinetics, the experimental data were analyzed using several kinetic models, including the pseudo-first-order, pseudo-second-order, Elovich, Langmuir kinetics and intraparticle diffusion models. A detailed description of the models can be found in the supporting information.

### Statistical analysis

Three replicates were used for all analyses. One-way analysis of variance was performed with OriginPro 8 (*p* < 0.05) performed. The data are shown as an average with a standard deviation. Statistical parameters, such as the coefficient of determination (R^2^, were explored to evaluate the performance of model.

## Results and discussion

### Characterization of amine-functionalized lignin and unmodified lignin

**Fig. 2:**
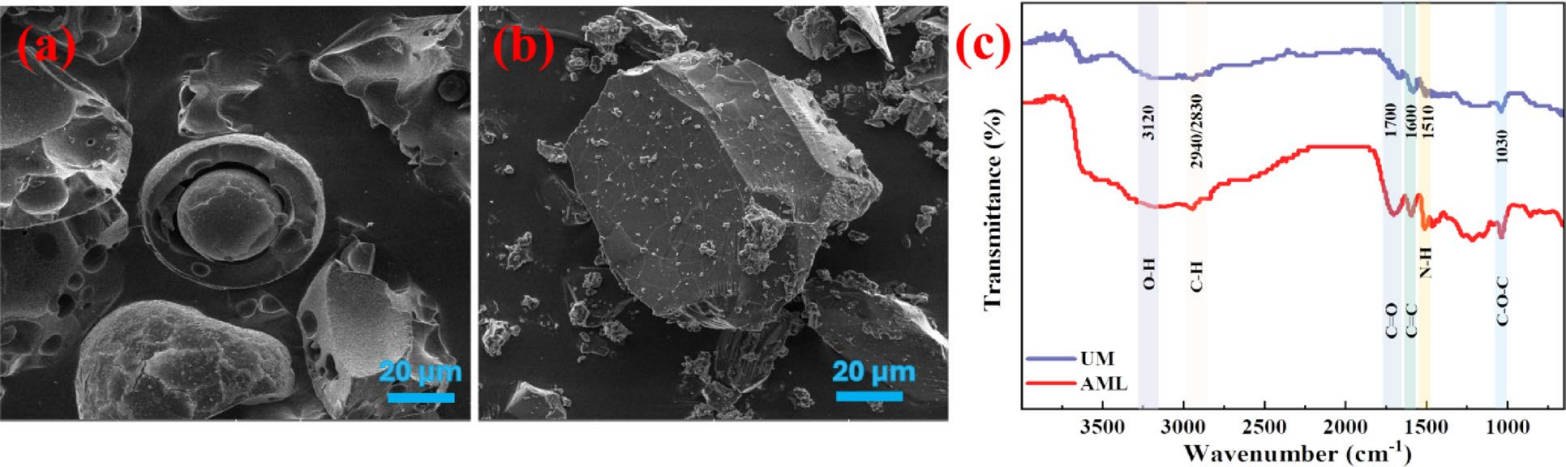
SEM images of (**a**) unmodified lignin and (**b**) amine-functionalized lignin; (**c**) FTIR of unmodified lignin amine and amine-functionalized lignin.

**Fig. 3:**
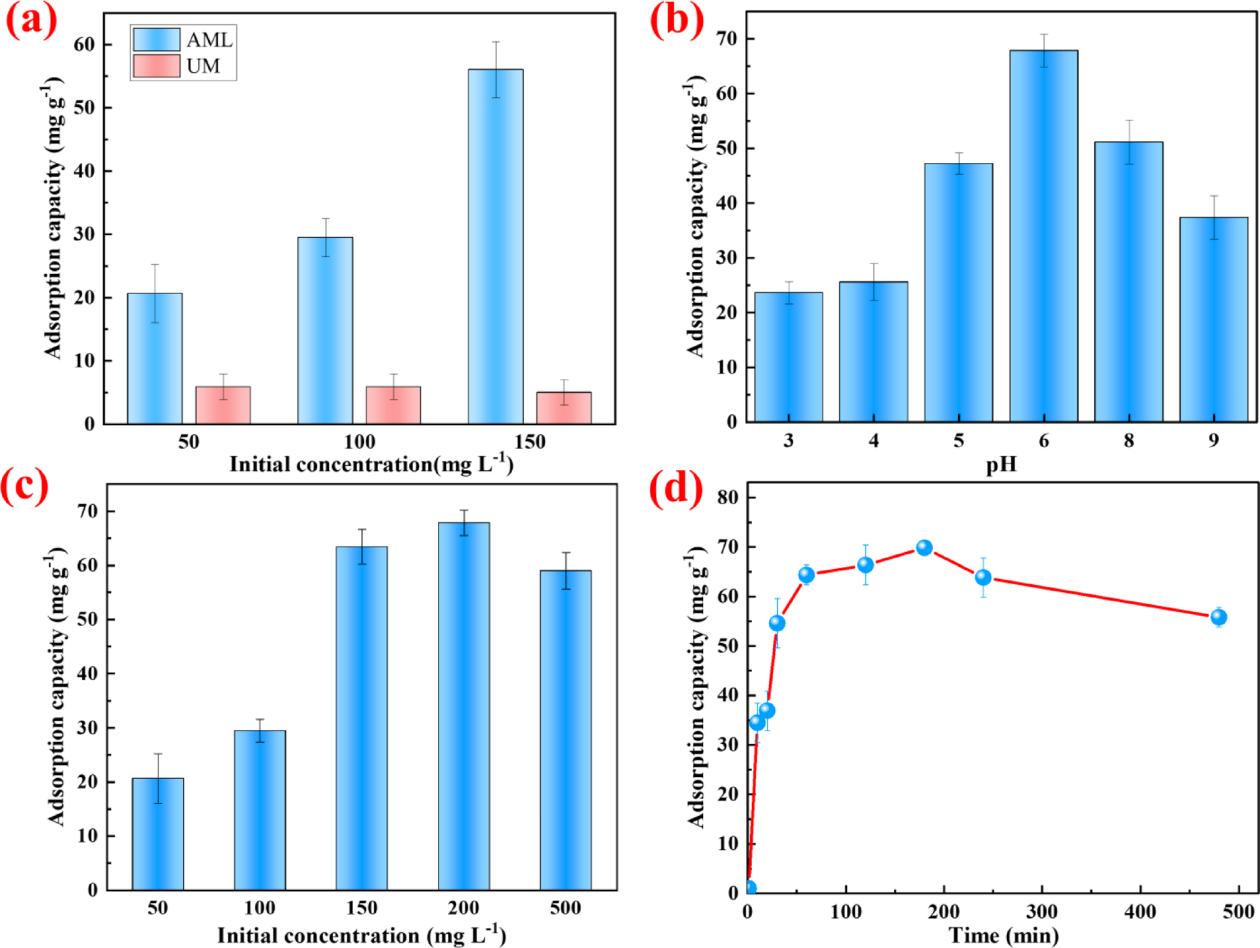
Effect of various factors for nitrate adsorption on AML (**a**) chemical amination (**b**) solution pH (**c**) initial concentration (**d**) contact time.

Various characterization techniques, including elemental analysis, FTIR, and SEM, have been employed to investigate the morphological and structural properties of amine-functionalized lignin and unmodified lignin, demonstrating the successful grafting of nitrogen into the lignin side chains through the chemical reaction depicted in Fig. 1.Elemental analysis was performed to determine the quantity of nitrogen in unmodified lignin (UM) and amine-functionalized lignin (AML). As shown in Table 1, the nitrogen, carbon and hydrogen contents significantly increased after functionalization, especially the nitrogen content, which increased from 0.209 to 2.345%. This is due to the amine functional groups being grafted onto the side chain of lignin^29^. In addition, the functional characteristics of lignin were determined through FTIR. As shown in Fig. 2, the FTIR spectrum of the UM sample before the reaction reveals a characteristic absorption band at 3120 cm^−1^, corresponding to the O–H stretching vibration. The peaks observed at 2940 cm^−1^ and 2830 cm^−1^ are attributed to C–H stretching vibrations. An absorption peak at 1600 cm^−1^ is assigned to the aromatic C=C skeletal vibration, while the peak at 1030 cm^−1^ corresponds to the C–O–C stretching vibration of methoxy groups^15^. After the reaction, the FTIR spectrum of the AML sample exhibits similar features. However, a new absorption band appears at 1700 cm⁻¹, which is assigned to the C=O stretching vibration, and a band at 1510 cm^−1^ is attributed to N–H stretching^30,31^. These changes indicate that the amine functional group was successfully grafted onto the lignin side while retaining its core aromatic structure. SEM micrographs of the amine-functionalized lignin and unmodified lignin are shown in Fig. 3. According to the SEM images, the morphology of lignin changed remarkably after chemical modification. The shape of the lignin was spherical, whereas primary, irregular particles tended to form aggregates after chemical modification. These differences in appearance indicated that lignin was dissolved and rebuilt during the Mannich reaction treatment^32–34^.

**Table 1:** Elementary analysis of sample materials.

Sample name	Chemical composites (%)		
	*N*	C	H
Unmodified lignin (UM)	0.209	45.621	1.691
Amine-functionalized lignin (AML)	2.345	57.085	2.539

### Batch adsorption studies

#### Comparison of the adsorption capacities of AML and UM

As shown in Fig. 3a the adsorption capacity of amine-functionalized lignin was significantly greater than that of unmodified lignin when the initial nitrate concentration ranged from 50 mg L^− 1^ to 150 mg L^− 1^. Specifically, the amine-functionalized lignin achieved a maximum adsorption capacity of 56.04 mg g^− 1^ for nitrate ions, whereas the unmodified lignin exhibited a lower adsorption capacity less than 10 mg g^− 1^. This performance can be attributed to the incorporation of amine groups through the Mannich reaction, which enhanced the interactions between the lignin-based adsorbent and nitrate ions, and highlighted the importance of functionalization in improving the adsorptive properties of lignin-based materials^34–36^. This comparison underscores the potential of amine-functionalized lignin as an effective and enhanced material for water treatment applications, particularly in mitigating nitrate pollution.

#### Effect of pH

The pH of a solution is a critical factor influencing the adsorption process. To determine the optimal pH for maximum nitrate adsorption capacity, a series of adsorption experiments were conducted across an initial pH range of 3–10 with a nitrate concentration of 100 mg L^− 1^. According to Fig. 3b, the adsorption capacity initially increased with an increase of pH and subsequently decreased as the pH continues to increase. A maximum adsorption capacity of approximately 65 mg g^− 1^ was observed at near pH of 6.2. Basically, lower pH conditions promote the protonation of surface amine groups, resulting in a positively charged surface that facilitated nitrate adsorption through electrostatic interactions^37^. However, when the pH dropped below 4.25, the adsorption capacity declined despite the presence of positively charged amine groups. This counterintuitive behavior was likely due to electrical double layer (EDL) compression and proton-dominated interfacial saturation^38^. Basically, at lower pH, the high concentration of H^+^ ions compress the EDL, thereby restricting the diffusion and effective approach of nitrate ions to the adsorbent surface and reducing electrostatic interaction efficiency. This similar electrostatic exclusion phenomenon has also been reported in other studies on nitrate removal under acidic conditions^39,40^. Furthermore, chloride ions (Cl⁻) introduced during pH adjustment with HCl may compete with nitrate for adsorption through weak anion exchange or contribute to ionic shielding near active sites^41^. In addition, the strongly acidic environment may partially dissociate surface functional groups, especially protonated amine moieties, which reduced the total number of accessible adsorption sites. Similar inhibitory effects of extremely low pH on anion adsorption have been observed in other functionalized biosorbent systems^14,15^. Conversely, at higher pH values, the surface amine groups of AML undergo deprotonation, reducing the positive charge and leading to electrostatic repulsion with nitrate ions, and this effect was further exacerbated by competition from excess OH^−^ ions at alkaline pH^41^. Nevertheless, residual adsorption capacity remained when the pH rase above 8, which consistent with reports that amine-functionalized adsorbents typically exhibit point of zero charge (pH_zpc_) values between 6.2 and 9.3. And it is implying that AML surfaces were positively charged below the pH_zpc_, favoring nitrate adsorption through electrostatic attraction^36,42–44^. Besides of electrostatic interactions, secondary mechanisms such as hydrogen bonding between uncharged amine groups (–NH_2_) and nitrate ions, as well as weaker dipole–dipole and donor–acceptor interactions, may also contribute to nitrate adsorption^44^. Overall, these results indicate that nitrate adsorption onto AML followed a pH-dependent mechanism that primarily governed by electrostatic attraction and additionally supported by secondary interactions such as hydrogen bonding.

#### Effect of initial adsorbate concentration

The relationship between the initial nitrate concentration and adsorption capacity highlights the key dynamics of the adsorption process, which is critical for optimizing adsorbent usage in practical applications and ensuring efficient contaminant removal without overloading the system. As shown in Fig. 3c, at lower nitrate concentrations, the active sites on the adsorbent were relatively unoccupied, and the increase of nitrate concentration generated a greater concentration gradient, serving as a driving force for mass transfer^45^. This enhanced driving force assisted nitrate ions to overcome resistance at the solid‒liquid interface, facilitating their rapid diffusion to the adsorbent surface and maximizing the utilization of available active sites^17^. However, as the nitrate concentration was over 150 mg L^− 1^ and continued to reach 500 mg L^− 1^, the adsorption capacity reached equilibrium, indicating that the active sites on the adsorbent surface have become saturated. Despite the higher nitrate anions concentration in solutions, the limited availability of unoccupied active sites prevented further adsorption, resulting in a plateau in the adsorption capacity. This plateau effect corresponded to the limitation of the adsorption capacity, which was consistent with the Langmuir isotherm model, where adsorption occurs at specific homogeneous sites and forms a single layer of adsorbed ions^46^. Additionally, as the nitrate concentration increased, more ions compete for a limited number of active sites, further highlighting the saturation of the adsorbent.

#### Effect of contact time

The contact time is a crucial parameter for evaluating the efficiency of adsorption processes, as it indicates the rate at which an adsorbent attains equilibrium with the adsorbate^47^. In this study, the effect of contact time on nitrate adsorption was examined. According to Fig. 3d, the adsorption equilibrium was achieved within 60 min, after that the adsorption rate decreased significantly. This slowdown can be attributed to the depletion of nitrate ions in the bulk solution and the reduced driving force for their diffusion into the surface of the adsorbent^15^. The rapid adsorption rate observed for nitrate onto the amine modified lignin indicated that the AML possessed a substantial number of accessible active sites and exhibited efficient mass transfer properties, facilitating the adsorption process^48^. Compared with other adsorbents, modified lignin has competitive performance. For example, similar results have been reported for chitosan-based materials, which also demonstrate rapid nitrate adsorption due to their functionalized amine groups^42,43^. In contrast, many conventional adsorbents often require longer contact times to achieve equilibrium because of the slower diffusion of nitrate ions into micropores and weaker interaction mechanisms^49^. For example, zeolite-based adsorbents may take several hours to reach equilibrium under similar experimental conditions. While activated carbon is highly effective for organic contaminants, its adsorption performance for anions such as nitrate is typically lower and slower, especially without surface modifications^6^. This rapid adsorption observed in the modified lignin highlights its potential for practical applications where time efficiency is critical, such as in continuous flow water treatment systems^17^.

### Adsorption isotherms and kinetics

#### Adsorption isotherms

**Fig. 4:**
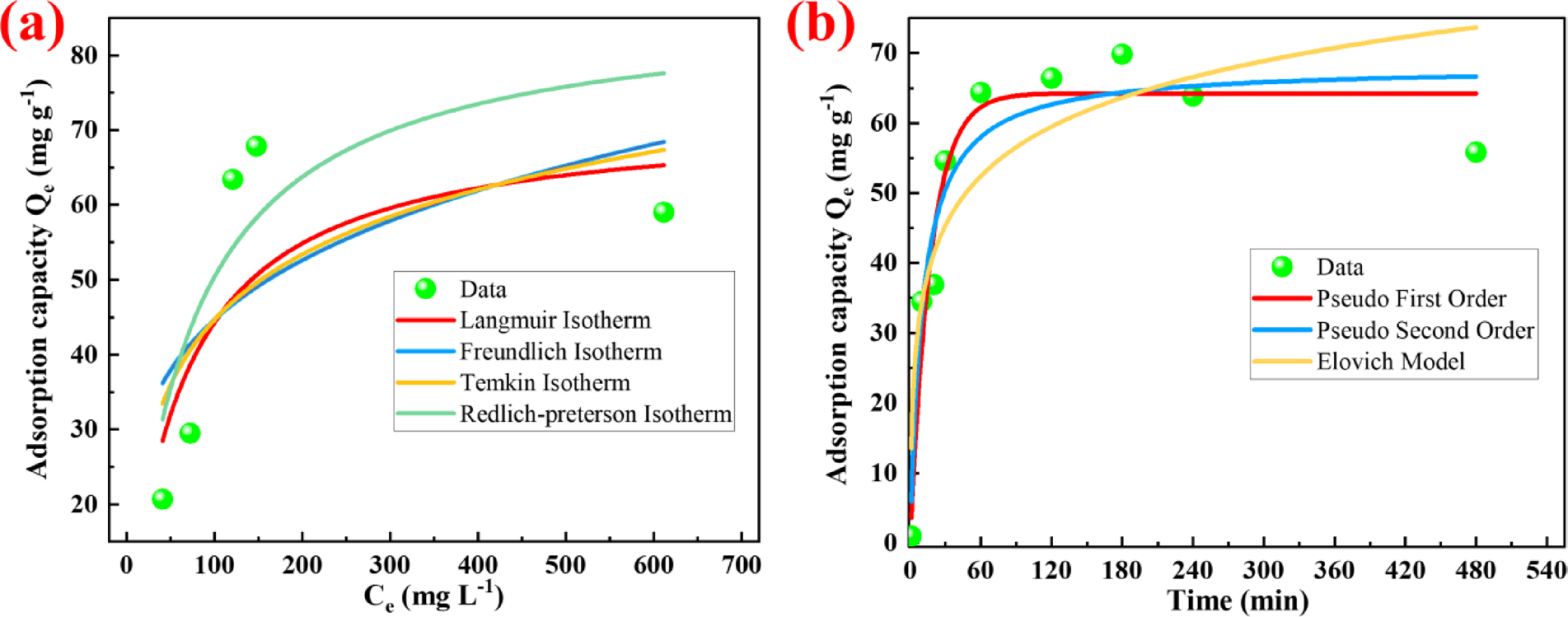
Adsorption isotherms (**a**) and kinetics (**b**) of AML for nitrate anions.

The adsorption behavior of nitrate ions onto amine-functionalized lignin (AML) was analyzed by various isotherm models, providing complementary insights into the process, as shown in Fig. 4a and Table 2. The Langmuir model assumes monolayer adsorption on a homogeneous solid surface where the active sites on adsorbent are uniformly distributed and each site binds to a single adsorbate ion, while the Freundlich model represents adsorption on heterogeneous surfaces with varying energy levels and affinities^50^. The R^2^ value of 0.940 yielded by the Langmuir model suggested that the nitrate adsorption process onto AML approximated monolayer coverage behavior on a relatively homogeneous surface. The theoretical maximum adsorption capacity Q_max_ was estimated to be 65.79 mg g⁻¹, reflecting the upper limit of nitrate uptake under the experimental conditions based on Langmuir assumptions^19^. Additionally, as shown in Fig. S1, the value of the separation factor R_L_ was less than 1, suggesting that the adsorption process was favorable under the experimental conditions^50^. On the other hand, the Freundlich model is empirical and represents adsorption on heterogeneous surfaces that adsorption sites have varying energy levels and affinities. The high R^2^ value of 0.995 obtained for the Freundlich model indicated that adsorption data were well described by this empirical equation and adsorption was not limited to uniform sites^50^. On a microscopic scale, the surface of AML may not be perfectly homogeneous. The energy levels of adsorption sites were influenced by the distribution of protonated amine groups (–NH_2_^+^) across the AML surface, and the strength of these electrostatic attractions between protonated amine groups and nitrate ions may vary depending on surface charge density and proximity of functional groups^37^. Also as shown in Table 2, the relatively high K_f_ value indicates strong affinity toward nitrate ions, likely driven by electrostatic attraction, while the Freundlich constant n being greater than 1 suggested that the adsorption process is favorable and intensifies with increasing nitrate concentration^51^. In addition to Langmuir and Freundlich models, the Temkin and Redlich–Peterson isotherm models were employed to further explore the adsorption behavior of nitrate onto AML. The Temkin model yielded a high correlation coefficient with R^2^ value of 0.993, suggesting that the adsorption heat decreases with increasing surface coverage^50^.This behavior consistent with electrostatical interation processes involving moderately heterogeneous surface energies as described in Freundlich model, which means the surface becomes increasingly occupied and the remaining binding sites exhibit lower affinities as the surface^50^. On the other hand, the Redlich–Peterson model showed a poor fit to the experimental data with R^2^ value of 0.551, which may suggests that the AML surface follow a relatively complex mechanism which may not be adequately described by a simple hybrid model combining homogeneous and heterogeneous adsorption assumptions^46^. Overall, based on aboved discussion of various isotherm models, these findings support an adsorption mechanism primarily governed by monolayer adsorption driven by electrostatic attraction, occurring on a moderately heterogeneous surface.

#### Adsorption kinetics

The adsorption kinetics of nitrate onto amine-functionalized lignin was systematically evaluated using several models, including the pseudo-first-order (PFO), pseudo-second-order (PSO) and Elovich models. As shown in Fig. 4b and Table 2, both the PFO and PSO models provided good fits to the experimental data, with correlation coefficients (R^2^) of 0.946 and 0.922, and calculated equilibrium adsorption capacities of 64.242 mg g^−1^ and 68.137 mg g^−1^, respectively. The pseudo-first-order model assumes that the rate of adsorption is proportional to the number of unoccupied sites^37^. The well fit with of PFO suggests that the process may incorporate rapid surface interaction, such as electrostatic attraction between nitrate and protonated amine groups, which also consistent with rapid initial adsorption due to the availability of highly active sites on the amine-functionalized lignin surface^18^. The pseudo-second-order model assumes that the rate is proportional to the square of the number of available adsorption sites^47^. The relatively high R^2^ value indicated that this process may also involve hydrogen bonding, where the protonated hydrogen acts as a donor and the oxygen in nitrate serves as an electron-rich acceptor^52^. In contrast, the poor fit of the Elovich model with R² value of 0.792 indicates that the adsorption process was not dominated in the assumptions of Elovich-type strong chemisorption with heterogeneous energy distribution^47^. To further investigate the rate-controlling mechanism, three additional models were applied, such as the modified intraparticle diffusion model, the Mathews and Weber model, and the Langmuir kinetic model. As shown in Fig. S2 and Table S1, the Langmuir kinetic model provided the best fit, suggesting that the rate-limiting step in the later stage was associated with the progressive saturation of active surface sites, ultimately leading to equilibrium^53^. Despite a moderately good correlation with R^2^ value of 0.924 was observed for the modified intraparticle diffusion model, the poor fit of the Mathews–Weber model suggested that intraparticle diffusion was not the only dominant rate-limiting step in the whole adsorption process^53^.

**Table 2:** Best-fit parameters of the adsorption isotherm and kinetic models.

	Parameter 1	Parameter 2	*R* ^2^
Langmuir model	K_L_ = 0.016 ± 0.000	Q_max_ = 65.794 ± 0.409	0.940
Freundlich model	K_F_ = 15.102 ± 14.209	*n* = 2.1 ± 0.409	0.995
Temkin	$\:{K}_{T}$ = 12.532 ± 0.032	A = 0.354 ± 0.004	0.993
Redlich-Peterson	$\:{K}_{RP}$ = 1.201 ± 2.44	$\:{a}_{RP}$ = 0.014±0.099	0.551
Pseudo-first order	k_1_ = 0.057 ± 0.009	q_e_ = 64.242 ± 2.556	0.946
Pseudo-second order	k_2_ = 0.001 ± 0.001	q_e_ = 68.137 ± 3.917	0.922
Elovich model	A = 28.210 ± 31.190	B = 0.098 ± 0.025	0.792

### Performance comparison with other amine-functionalized bioadsorbents

To better assess the adsorption performance of the amine-functionalized lignin (AML) developed in this study, Table 3 provides a comparative overview of reported amine-functionalized bio-based adsorbents with considering key operational parameters, such as maximum adsorption capacity (Q_max_), equilibrium time, and effective pH range. The results show that AML exhibits competitive performance under environmentally relevant conditions, indicating its suitability for practical nitrate remediation. While the Q_max_ of AML (65.79 mg g^−1^) is lower than that of some highly engineered materials, such as PEI-grafted cellulose (232.56 mg g^−1^) and chemically activated tea waste (up to 136.43 mg g^−1^), this difference can be attributed to the relatively mild and environmentally friendly functionalization strategy adopted in this work. Specifically, AML was synthesized through a one-pot urea–formaldehyde Mannich reaction, which introduces primary amine groups without relying on toxic organic solvents, intensive crosslinking, or nanoparticle incorporation. In contrast, adsorbents with higher Q_max_ often require multi-step syntheses involving hazardous chemicals, e.g., epichlorohydrin, DMF, high energy input, or complex post-treatment steps, which increase cost and environmental burden^4,9,15,54^. Despite its moderate adsorption capacity, AML offers a desirable trade-off between efficiency, environmental compatibility, and scalable preparation route, which underscores its promise as a cost-effective and eco-friendly solution for nitrate removal in sustainable water treatment applications.

**Table 3:** Comparative table with other amine functionalized bio-based adsorbents towards nitrate.

Adsorbent	Dosage (g L^− 1^)	pH	Contact time (min)	Temperature (℃)	Maximum adsorption capacity (Q_max_)	Concentration range (mg L^− 1^)	References
Cell-g-E/PEI	1	6	240	30	232.56	1–40	^7^
Amine-crosslinked tea waste (ACTW)	2	6	60	25	136.43	25-1500	^39^
Amine grafted chitosan	2	7	60	30	38.40	20–140	^42^
Amine-grafted agriculture wastes	4	6.5	Not mentioned	30	43.75–63.25	1–30	^55^
Amine-graft coconut copra	1	6.5	Overnight	25	59.20	20	^2^
Amine modified wheat straw (AMWS)	0.5	6.8	120	25	73	700–1700	^41^
Modified wheat residue (MWS)	2.5	6.8	150	25	85	50–500	^56^
Amine graft lignin (AML)	0.625	6.8	120	25	65.79	50–500	This work

## Proposed adsorption mechanism for amine-functionalized lignin-based adsorbents

**Fig. 5:**
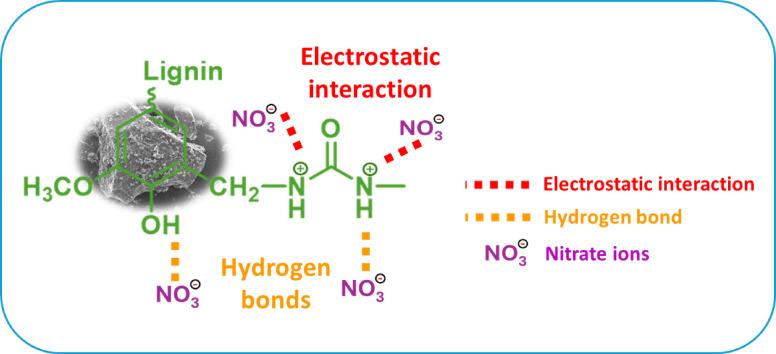
Schematic diagram of the adsorption interactions between amine-functionalized lignin and nitrate anions in aqueous solutions.

The adsorption mechanism of amine-functionalized lignin-based adsorbents for nitrate ions is driven by a synergistic combination of primarily electrostatic attractions and supplementary contributions from hydrogen bonding facilitated by the chemical structure and properties of the amine-functionalized material, as shown in Fig. 5. The introduction of amine groups through the Mannich reaction provided positively adsorption sites to the adsorbent, especially under acidic to neutral pH conditions, where the amine groups are protonated to form –NH_2_^+^. These positively charged sites strongly attract and bind negatively charged nitrate ions through electrostatic forces, which serve as the primary adsorption mechanism. Hydrogen bonding also plays a critical role, as the hydrogen atoms in the protonated amine groups can form bonds with the oxygen atoms in nitrate ions, providing additional stabilization during the adsorption process. At the same time, inherent phenolic (–OH) groups of lignin may also contribute to hydrogen bonding with nitrate ions, though these interactions are weaker compared to those involving protonated amines. To further elucidate the adsorption behavior and surface characteristics, isotherm analyses were performed. The adsorption data fit relatively well with the Freundlich, Temkin, and Langmuir models, whereas the Redlich–Peterson model showed poor fitting, which indicating that nitrate adsorption is controlled by a combination of monolayer adsorption and surface heterogeneity effects, driven mainly by electrostatic interactions, consistent with a complex adsorption mechanism involving both homogeneous and heterogeneous characteristics. The kinetic fitting results of the adsorption process support the proposed mechanism that the Langmuir kinetics model showing the best fit to the experimental data, which suggests that the rate-limiting step in the adsorption process is controlled by the adsorption site occupation. This was further supported by the rapid attainment of adsorption equilibrium, suggesting that nitrate uptake was governed mainly by surface-controlled mechanisms, particularly through the progressive occupation of amine-functional sites that were driven by electrostatic interactions with nitrate anions. Although adsorption experiments were conducted at only a single temperature (25 °C), thermodynamic insights can still be inferred through comparison with similar systems. Literature reports ΔH° values range between − 4.52 kJ/mol and − 13.9 kJ/mol for nitrate adsorption onto amine-functionalized biosorbents, which is indicative of weak chemisorption^7,37,42,44^. These findings are consistent with the interaction mechanisms proposed and support the inference that nitrate adsorption onto AML was primarily exothermic and dominated by electrostatic attraction, supplemented by hydrogen bonding related secondary mechanism. Together, these findings indicate that nitrate adsorption onto AML was governed by a combination of monolayer adsorption and surface heterogeneity effects, primarily driven by electrostatic attraction, hydrogen bonding, and complementary interactions with amine functional groups, rather than pore-filling physical adsorption. Those findings highlight the critical role of amine functionalization in enhancing adsorption performance of the material and nitrate removal.

## Conclusion and future perspectives

This study successfully demonstrated the efficacy of biowaste-based adsorbents synthesized through the Mannich reaction for the enhanced removal of nitrate ions from water. By incorporating amine groups into the lignin structure, the adsorbent demonstrated significant nitrate adsorption capacity under a range of experimental conditions, including varying contact times, pH levels, and initial nitrate concentrations. Structural and functional modifications were confirmed through FTIR, SEM, and elemental analysis, ensuring the successful grafting of amine groups onto lignin. The maximum adsorption capacity of 65.79 mg g^− 1^ was determined via the Langmuir isotherm, with a rapid adsorption rate occurring within 1 h. The adsorption kinetics were primarily governed by the activation of adsorption sites, with the process being driven by electrostatic interactions between the amine-functionalized lignin and nitrate ions. These findings suggest that these biowaste-based adsorbents offer a cost-effective, environmentally friendly solution for mitigating nitrate pollution in water bodies, contributing to sustainable water treatment and environmental protection efforts. As a direction for future research, a comprehensive evaluation of the reusability of adsorbent through multiple adsorption-desorption cycles are essential, alongside an investigation of the effects of competing anions in realistic water matrices to better assess practical viability and selectivity. It is also recommended to extend this study beyond synthetic solutions to include real groundwater and surface water samples contaminated with nitrate.

## Supplementary Information

Below is the link to the electronic supplementary material.

Supplementary Material 1

## Data Availability

All data generated or analyzed during this study are included in this published article. For additional data requests, please contact Tiantian Li at tiantianli121@126.com.
