# Supplementary material for: Enhanced nitrate removal from aqueous solutions using amine-functionalized biowaste-derived adsorbent
Source: Sci Rep. 2025 Oct 21;15:36534. doi: 10.1038/s41598-025-17259-9 (PMC12540652; doi:10.1038/s41598-025-17259-9)
Supplement: Supplementary file 1 — Supplementary Material 1 [file 41598_2025_17259_MOESM1_ESM.docx]

**Supporting information**

For adsorption isotherm study, the nonlinearized form of the Langmuir model is given by the following equations:

$Q_{e}=\frac{q_{m}K_{L}C_{e}}{1+K_{L}C_{e}}$ [S1]

where K_L_ is the Langmuir isotherm constant (L mg^-1^) and q_m_ is the maximum monolayer coverage capacity (mg g^-1^).

The separation factor (R_L_) is calculated by the following equation: $R_{L}=\frac{1}{1+K_{L}C_{0}}$ [S2], where if R_L_ >1, the adsorption is unfavorable; if R_L_ =1, it is linear; and if R_L_ < 1, the adsorption is favorable.

The nonlinear form of the Freundlich model is given by the following equation:

$Q_{e}=K_{F}C_{e}^{1/n}$ [S3]

where K_F_ is an indicator of the adsorption capacity. The higher the maximum capacity is, the greater the K_F;_ n denotes the heterogeneity factor related to the adsorption intensity.

The nonlinear forms of the Temkin isotherm model are given by the following equation:

$Q_{e}={\frac{RT}{b}*In(AC}_{e})$ [S4]

where $K_{T}$=$\frac{RT}{b}$, R is 8.314(J/(mol K)), T is the temperature (K), b is the Temkin constant (J mol^-1^), A is Temkin isotherm constant (L g^-1^).

The nonlinear form of the Redlich-Peterson isotherm model is given by the following equation:

$Q_{e}= \frac{K_{RP}C_{e}}{1+a_{RP}C_{e}^{n}}$ [S5]

where $K_{RP}$ (L g^-1^), $a_{RP}$ ( L^g^  mg ^-g^ ) are constants, n is the exponent (0 ≤ n ≤1). We can see from S4 that when n equals to 1, the R-P model reduces to the Langmuir model and when n equals to 0 or Ce approaches to 0, it will reduce to the linear model. Furthermore, if $C_{e}$ approaches to infinite, $Q_{e}$which reduces to the Freundlich model

For the adsorption kinetics study, the pseudo-first-order, pseudo-second-order and intraparticle diffusion models were used to evaluate the kinetic experimental data.

The nonlinear form of the pseudo-first-order model was described below:

$q_{t}=q_{e}\left( 1- e^{k_{1}t} \right)$ [S6]

The nonlinear form of the pseudo-second-order model was as follows:

$q_{t}=\frac{{q_{e}}^{2}k_{2}t}{1+k_{2}t}$ [S7]

where $q_{e}$ (mg g^-1^) and $q_{t}$ (mg g^-1^) are the amounts of nitrate adsorbed by the amine-functionalized lignin adsorbent at equilibrium and at different time intervals, respectively; $k_{1}$ (min^-1^) and $k_{2}$ (g mg^-1^ min) are the pseudo-first-order and pseudo-second-order rate constants, which are used to describe the rate of adsorption equilibrium; and h represents the initial adsorption rate (mg g^-1^ min).

The modified interparticle diffusion model was given as follows:

$q_{t}=k_{p}t^{0.5}$ (0≤ t ≤ t_1_) [S8]

$q_{t}-q_{t-1}=k_{2}\left( t- t_{1} \right)^{1/2}$ (t_1_≤ t ≤ t_2_) [S9]

where $k_{1}$ and $k_{2}$ are the intraparticle diffusion rate constant (mg g^-^1 min^0.5^).

The nonlinear form of the Elovich kinetics model was given by the following equation:

$q_{t}=\frac{1}{b}ln\left( 1+abt \right)$ [S10]

Where a is initial adsorption rate, b is constant

The adsorption capacity of nitrate ions by either amine-functionalized lignin (AML) or unmodified lignin (UM) was calculated followed by equations in supporting information

$Q_{e}= \frac{\left( C_{0}-C_{e} \right)\times V}{m}$ [S11]

where C_0_ (mg L^-1^) is the initial concentration of nitrate and C_e (_mg L^-1^) is the nitrate concentration at equilibrium; V(L) is the volume of the adsorption; m (g) is the mass of the amine-functionalized lignin or unmodified lignin; and Q_e_ (mg g^-1^) is the adsorption capacity of nitrate at equilibrium.

The external adsorption process was modeled using the Mathews and Weber model, which can be described as follows:

$q_{t}=\frac{C_{0}}{m_{s}}\left( \text{1}{-\text{e}}^{-k_{\text{M\&W}}St} \right)$ [S12]

Where *q*_t_ (mg/g) is the adsorption capacity, *C*_0_ (mg/L) is the initial concentration of the adsorbate, *m*_s_ (g/L) is the mass of adsorbent per unit volume of solution (g/L), *k*_M&W_*S* (1/h) is the model constant.

The Langmuir kinetics model can represent the monolayer chemical adsorption process, and it was employed to model the kinetics of adsorption onto activated sites. The Langmuir kinetics model is described by Eq. (S17).

$\frac{dq_{t}}{dt}=k_{a}C_{t}\left( q_{e}-q_{t} \right)-k_{d}q_{t}$ [S13]

Where *C*_t_ (mg/L) is the concentration of the adsorbate at time *t*, *k*_a_ L/(mg·h) is the adsorption rate constant, *k*_d_ (1/h) is the desorption constant, *q*_e_ (mg/g) is the equilibrium adsorption capacity.

**Figure S1.** The separation factor (R_L_) of AML for nitrate anions.

**
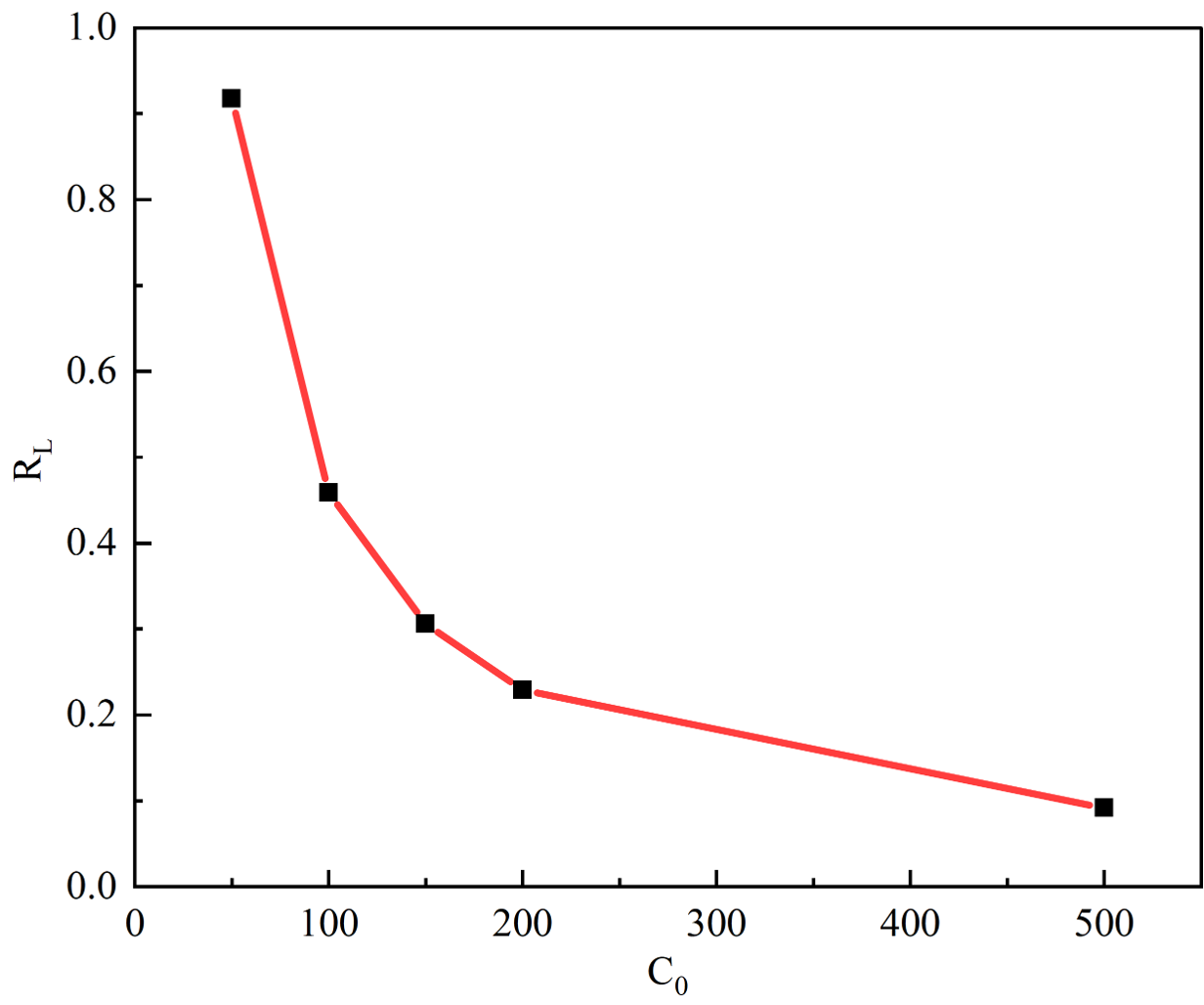
**

**Figure S2.** The fitting curves of the modified intraparticle diffusion model, the Mathews and Weber model, and the Langmuir kinetics model.


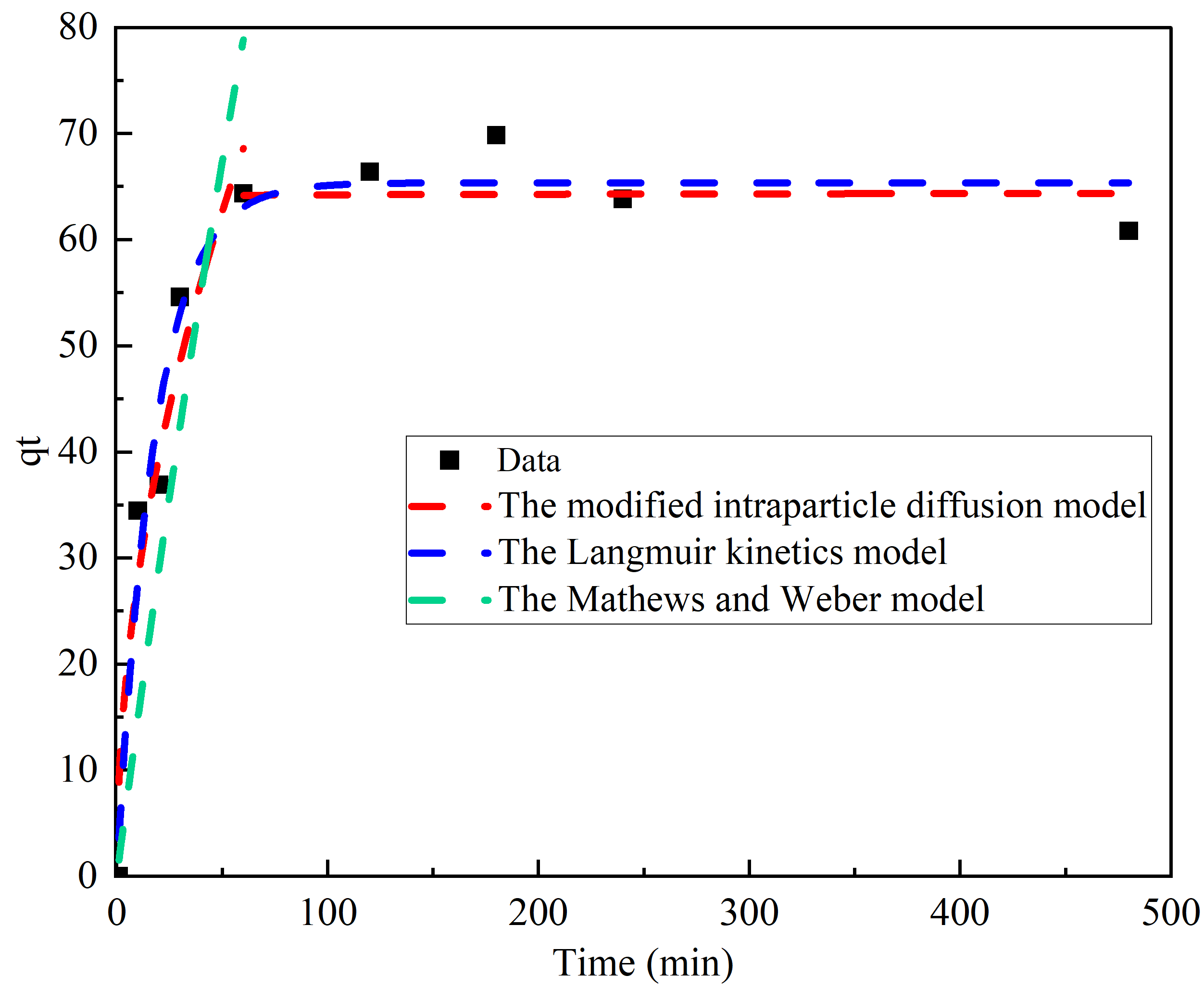


**Table S1.** The fitting results of the modified intraparticle diffusion model, the Mathews and Weber model, and the Langmuir kinetics model.

|  | Parameters | R^2^ |
| --- | --- | --- |
| Langmuir kinetic model | $k_{a}$ = 0.0002; $k_{d}$ = 0.0108; $q_{e}$ = 81.477 | 0.963 |
| Modified intraparticle diffusion model | $k_{1}$= 0.856; $k_{2}$=0.01 | 0.924 |
| Mathews and Weber model | $k_{MWS}$=0.00508 | 0.667 |
